# Neuroinflammation and status epilepticus: a narrative review unraveling a complex interplay

**DOI:** 10.3389/fped.2023.1251914

**Published:** 2023-11-21

**Authors:** T. Foiadelli, A. Santangelo, G. Costagliola, E. Costa, M. Scacciati, A. Riva, G. Volpedo, M. Smaldone, A. Bonuccelli, A. M. Clemente, A. Ferretti, S. Savasta, P. Striano, A. Orsini

**Affiliations:** ^1^ Clinica Pediatrica, Fondazione IRCCS Policlinico San Matteo, Pavia, Italy; ^2^ Pediatric Neurology, Pediatric Department, AOUP Santa Chiara Hospital, Pisa, Italy; ^3^ Pediatric Oncology, Pediatric Department, AOUP Santa Chiara Hospital, Pisa, Italy; ^4^ Pediatric Department, AOUP Santa Chiara Hospital, Pisa, Italy; ^5^ Pediatric Neurology and Muscular Diseases Unit, Department of Neurosciences, Rehabilitation, Ophthalmology, Genetics, Maternal, and Child Health, IRCCS Istituto “G. Gaslini”, Genoa, Italy; ^6^ Department of Neurosciences, Rehabilitation, Ophthalmology, Genetics, Maternal and Child Health, University of Genoa, Genoa, Italy; ^7^ Pediatrics Unit, Neuroscience, Mental Health and Sense Organs (NESMOS) Department, Faculty of Medicine and Psychology, Sapienza University of Rome, Rome, Italy; ^8^ Pediatric Clinic and Rare Disease Microcitemico Hospital, University of Cagliari, Cagliari, Italy

**Keywords:** epilepsy, status epilepticus, neuroinflammation, inflammation, cytokines

## Abstract

Status epilepticus (SE) is a medical emergency resulting from the failure of the mechanisms involved in seizure termination or from the initiation of pathways involved in abnormally prolonged seizures, potentially leading to long-term consequences, including neuronal death and impaired neuronal networks. It can eventually evolve to refractory status epilepticus (RSE), in which the administration of a benzodiazepine and another anti-seizure medications (ASMs) had been ineffective, and super-refractory status epilepticus (SRSE), which persists for more than 24 h after the administration of general anesthesia. Objective of the present review is to highlight the link between inflammation and SE. Several preclinical and clinical studies have shown that neuroinflammation can contribute to seizure onset and recurrence by increasing neuronal excitability. Notably, microglia and astrocytes can promote neuroinflammation and seizure susceptibility. In fact, inflammatory mediators released by glial cells might enhance neuronal excitation and cause drug resistance and seizure recurrence. Understanding the molecular mechanisms of neuroinflammation could be crucial for improving SE treatment, wich is currently mainly addressed with benzodiazepines and eventually phenytoin, valproic acid, or levetiracetam. IL-1β signal blockade with Anakinra has shown promising results in avoiding seizure recurrence and generalization in inflammatory refractory epilepsy. Inhibiting the IL-1β converting enzyme (ICE)/caspase-1 is also being investigated as a possible target for managing drug-resistant epilepsies. Targeting the ATP-P2X7R signal, which activates the NLRP3 inflammasome and triggers inflammatory molecule release, is another avenue of research. Interestingly, astaxanthin has shown promise in attenuating neuroinflammation in SE by inhibiting the ATP-P2X7R signal. Furthermore, IL-6 blockade using tocilizumab has been effective in RSE and in reducing seizures in patients with febrile infection-related epilepsy syndrome (FIRES). Other potential approaches include the ketogenic diet, which may modulate pro-inflammatory cytokine production, and the use of cannabidiol (CBD), which has demonstrated antiepileptic, neuroprotective, and anti-inflammatory properties, and targeting HMGB1-TLR4 axis. Clinical experience with anti-cytokine agents such as Anakinra and Tocilizumab in SE is currently limited, although promising. Nonetheless, Etanercept and Rituximab have shown efficacy only in specific etiologies of SE, such as autoimmune encephalitis. Overall, targeting inflammatory pathways and cytokines shows potential as an innovative therapeutic option for drug-resistant epilepsies and SE, providing the chance of directly addressing its underlying mechanisms, rather than solely focusing on symptom control.

## Introduction

According to the International League Against Epilepsy (ILAE), Status epilepticus (SE) is a condition resulting either from the failure of the mechanisms responsible for seizure termination or from the initiation of mechanisms which lead to abnormally prolonged seizures. It is a condition that can have long-term consequences, including neuronal death, neuronal injury, and alteration of neuronal networks, depending on the type and duration of seizures (1). Based on the clinical presentation, SE can be classified into convulsive SE (featured by motor symptoms and impairment of consciousness), and non-convulsive SE (2). Given the severity of SE and the potential development of irreversible brain damage, there is an urgent need to dissect its pathogenesis to find new potential therapeutic targets. Specifically, the most relevant therapeutic challenges are represented by refractory status epilepticus (RSE), in which the administration of a benzodiazepine bolus and another anti-seizure medication (ASM) does not resolve the clinical picture (3), and super-refractory status epilepticus (SRSE), which persists for more than 24 h after the administration of general anesthesia (2).

During the last years, an increasing interest has been posed on the involvement of neuroinflammation in epileptogenesis and in the pathogenesis of developmental and epileptic encephalopathies (4). Neuroinflammation is also implicated in enhancing and maintaining the pathogenic mechanism of SE. Therefore, the use of drugs acting on the inflammatory response (especially, anti-cytokine agents) has been empirically introduced in patients with RSE to achieve seizure control, while preclinical studies have focused on the identification of potential targets to regulate neuroinflammation in epilepsy and SE.

In this paper, we will present the main molecular mechanisms responsible for neuroinflammation in SE and the effect of currently available therapeutic strategies for convulsive SE on the inflammatory response. Furthermore, the potential new therapeutic agents targeting neuroinflammation will be presented, focusing on data deriving from preclinical and clinical studies.

## Materials and methods

In this narrative review, we performed comprehensive analysis of existing literature through three reputable databases: PubMed, Embase, and Cochrane. Our search was carefully guided by a thoughtfully curated set of keywords, designed to ensure a thorough investigation of pertinent research. These keywords included terms such as “epilepsy,” “inflammation,” “neuro-inflammation,” “status epilepticus,” “FIRES,” “NORSE,” “RSRE,” and “SRSE.” To maintain consistency, we specifically focused on articles published in the English language.

Our inclusion criteria were designed to encompass a wide spectrum of studies that delved into various facets of the relationship between status epilepticus (SE) and neuroinflammation. This included both clinical and preclinical investigations, studies exploring the role of cytokines and biomarkers, and an examination of how current and future SE therapies intersect with inflammatory pathways. Studies that exclusively concentrated on clinical aspects of SE or those that did not directly contribute to our primary research question were excluded.

To ensure a rigorous and methodical approach to our review, two independent reviewers conducted an initial screening by evaluating study titles and abstracts. This initial screening aimed to assess the relevance of each study to our research question. Subsequently, full-text articles of potentially relevant studies underwent a more detailed review to determine their suitability for inclusion in our narrative review. In instances where differences or uncertainties arose during the review process, a third reviewer was engaged to facilitate discussion and achieve consensus. This collaborative effort was instrumental in maintaining the quality and consistency of study selection throughout our review process.

## Results

### Neuroinflammatory mechanisms involved in status epilepticus

The main results of our research are listed in Table 1. SE can lead to significant morbidity and mortality, and the mechanisms underlying its development and progression are complex and not fully understood. However, several studies suggest that inflammation plays a significant role in the pathogenesis of SE.

**Table 1:** Overview of the main clinical studies involving monoclonal antibodies targeting neuroinflammation in status epilepticus.

Author	Year	Patients (*n*)	Population	Treatment	Outcome
Lai et al. (151)	2020	25	FIRES	Anakinra	Reduction of the seizure frequency >50% in more than half of the patients. 3/25 died during treatment
Sa et al. (152)	2019	2	FIRES	Anakinra	P1: Transient improvement and resolution of SE, with relapse after discontinuation P2: No clinical improvement
Shrestha et al. (154)	2023	6	FIRES	Anakinra	Reduction of the seizure frequency in all the analyzed patients Progression of neurocognitive impairment in 3 patients
Aledo-Serrano et al. (153)	2022	5	FIRES	Anakinra (5 patients)	3/5 experienced a 20%–50% reduction in seizure frequency
				Tocilizumab (1 patient)	30% reduction in seizure frequency
Girardin et al. (155)	2023	2	FIRES/NORSE	Tocilizumab	P1: Resolution of SE and improved neurocognitive outcome. P2: Transient resolution of SE, with relapse of seizures at discontinuation. Persistence of neurocognitive impairment, despite improvement
Jun et al. (132)	2018	7	NORSE	Rituximab	Persistence of SE. Patients eventually received Tocilizumab
				Tocilizumab	SE was resolved in 6/7 patients
Cantarin-Extremera et al. (133)	2020	2	NORSE	Tocilizumab	Decrease in the seizure frequency in both patients

SE, status epilepticus; FIRES, febrile infection-related epilepsy syndrome; NORSE, new-onset refractory status epilepticus.

Higher levels of pro-inflammatory cytokines, such as interleukin-1 beta (IL-1β), interleukin-6 (IL-6), and tumor necrosis factor-alpha (TNF-α), have been reported in SE (4). These molecules can be secreted by activated glial cells, such as microglia and astrocytes, and can be associated with the release of pro-inflammatory molecules by damaged neurons, including high-mobility group box 1 protein (HMGB1) and damage-associated molecular patterns [DAMPs (5)]. This molecular cascade has been suggested to trigger glia-mediated neuroinflammation. On the other hand, different authors have suggested that changes in neuronal activity and energy metabolism caused by SE could also activate microglia and astrocytes. For example, prolonged seizures can induce an increase in extracellular potassium, which eventually lead astrocytes to release neurotransmitters that, in turn, can modulate synaptic activity (6). Seizures may also directly activate microglia and astrocytes by triggering receptors on their cell surfaces. For instance, seizures activate the P2X7 receptor of microglia, leading to the release of pro-inflammatory cytokines (7).

Activated microglia and astrocytes have been shown to express IL-1β (8), which might promote seizures through the upregulation of NMDA receptors on postsynaptic cells (9). Interestingly, the IL-1β antagonist, Anakinra, has been shown to ameliorate long-term potentiation impairment (10). Furthermore, preclinical studies have showed that TNF-α is also released by activated glial cells. It has been reported to regulate N-cadherin (11), which in turn plays a pivotal role in modulating the organization of excitatory and inhibitory synapses. Moreover, TNF-α could potentially promote seizures by increasing microglial glutamate release through the upregulation of glutaminase enzyme (12), as well as by enhancing the expression of AMPA receptors (13). Finally, it has also been shown that TNF-α affects inhibitory neurotransmission by promoting GABA receptor endocytosis (14).

Astrocytes and microglia could also increase the levels of IL-6 within the central nervous system (CNS), which eventually reduce long-term potentiation and hippocampal neurogenesis, while promoting gliosis. These effects could contribute to creating a subset for epilepsy (15).

The blood-brain barrier (BBB) is a critical component of the immune control within the brain, moduling the access of immune cells and cytokines. In SE, the BBB might become dysfunctional, allowing immune cells and cytokines to access the brain more easily. Such disruption eventually participates in the development of neuroinflammation and neuronal damage (16). Inflammatory mediators, namely IL-1, IL-6, and TNF alpha, can contribute to the BBB breakdown, leading to increased permeability and leukocyte infiltration (17). Increased levels of white blood cells into the hippocampus, such as neutrophils, have been associated with neurodegeneration and temporal lobe epilepsy (18). Moreover, it has been hypothesized that the upregulation of adhesion molecules promoted by seizures, such as VCAM-1 and CD44, might contribute to BBB permeability, neuro-inflammation, and subsequent seizure generation (19).

Inflammatory response in SE can also lead to the activation of the **complement** system, a key component of the immune response. Notably, the activation of C1q-C3 signaling pathway has been observed in animal models and in humans with SE (20). The activation of the complement system can concur to the recruitment of immune cells and the development of inflammation in the brain.

Finally, the activation of the inflammasome, which plays a pivotal role in innate immune response, has also been implicated in the development of SE. In fact, the inflammasome is activated in response to danger signals, such as those produced during seizures, leading to the release of IL-1β (5). In animal models of SE, blocking inflammasome activation has been proved to reduce seizure activity and improve epilepsy outcomes (21).

In addition to the excitatory processes mentioned earlier, inhibitory neurotransmission appears to play a significant role in the complex inflammatory mechanisms associated with the development of epilepsy. Notably, elevated levels of IL-1β have been correlated with a reduced GABA-A currents in cases of temporal lobe epilepsy. Furthermore, TNF-α has been observed to promote the endocytosis of GABA receptors.

Nonetheless, the role of anti-inflammatory molecules has been increasingly studied during the last years, including COX-2-selective and nonselective inhibitors (22, 23).

These findings underscore the intricate interplay between inflammatory cytokines and the regulation of inhibitory neurotransmission in the pathogenesis of epilepsy. Understanding these multifaceted processes holds promise for advancing our knowledge of epilepsy development and potentially uncovering new avenues for therapeutic intervention.

### Preclinical and clinical evidences on neuroinflammation and status epilepticus

#### Preclinical studies

Preclinical studies with transgenic murine models clearly show that neuroinflammation can promote the generation and recurrence of seizures by increasing neuronal excitability and therefore lowering seizure threshold (13, 22, 24). For instance, overexpression of caspase-1 in subicular pyramidal neurons was sufficient to induce pharmacoresistant temporal lobe epilepsy in rats (25). On the other hand, preventing activation mediated by the IL-1 receptor 1 (IL-1R1)/TLR4 signaling pathway both pharmacologically or via genetic intervention, leads to a decrease in both acute and chronic seizure frequency (26). IL-1R1 co-localizes with the N-methyl-d-aspartate (NMDA) receptor, which is involved in excitotoxicity and seizures (27–29). Induction of the IL-1β/IL-1R1 cascade causes post-translational modifications in NMDAR, resulting in NMDA-induced Ca2 + influx, neuronal activation, and hippocampal kainate (KA) seizures in mice (9, 30, 31). HMGB1 activation of TLR4 exerts similar effects (32) and TLR4 KO mice show reduced KA-induced seizure activity (33). TNF-α also plays a pro-inflammatory role and mice with excessively high levels of this cytokine are at high risk of epilepsy (34). However, this signaling cascade plays a dual role in seizure susceptibility in mice, depending on whether TNF-α acts through TNFR1 or 2 (35). While TNFR1 KO mice show a decreased incidence of seizure, TNFR2 KO mice display increased seizure behavior (36).

Glial cells surrounding neurons in the brain are the main source of these inflammatory cytokines and their functions affect neuroinflammation and susceptibility to seizures. Microglia are a CNS-resident macrophage population which can have inflammatory properties during infection or sterile insult to the brain (37). The communication between microglia and neurons is important to maintain homeostasis and has been demonstrated to be neuroprotective in epilepsy. In fact, mice lacking the microglial P2Y2 receptor display worsened seizure behavior (38). Furthermore, inhibiting mTOR leads to delayed microglial activation in the hippocampus of mice with KA-induced epilepsy (39). Another type of glia, the astrocytes, perform metabolic, structural, homeostatic, and neuroprotective tasks in the CNS. One of their functions is to regulate extracellular glutamate levels through the glutamate uptake system. Inhibition of the astrocyte glutamate transporter GLT-1 in mice with cortical dysplasia lowers seizure threshold and enhances neuronal excitability (40). Furthermore, activation of the astrocytic TLR4-MyD88-ERK1/2 pathway in mice leads to over-excitation at the neuronal level and increased density of excitatory synapses following LPS injection, which could increase seizure susceptibility (41).

Interestingly, it is not always neuroinflammation that causes seizures, but also the other way around. Seizures can induce brain injury, which in turn can activate microglia and astrocytes to release a wide spectrum of inflammatory mediators with neurotoxic properties. High levels of inflammatory mediators can lead to enhanced neuronal excitation and BBB dysfunction in the mouse brain, ultimately causing drug resistance in epilepsy and recurrence of seizures, giving rise to a vicious cycle (42). Thus, understanding the unique molecular mechanisms of neuroinflammation in seizure disorders is crucial to identify inflammatory mediators and pathways which could act as biomarkers for development and severity of epilepsy, as well as becoming novel therapeutic targets. Pharmacological immunomodulation of these pathways might provide a novel avenue for treating epilepsy (42). For instance, it has been shown that inhibiting leukotriene D4 signaling attenuates seizure development in a murine model of chemically induced kindled seizure (43). Similar results were obtained by inhibiting IL-1β biosynthesis (44), as well as by administering an agonist of the prostaglandin E2 EP1 receptor and inhibiting COX-2 (45). Administration of different COX-2 inhibitors lead to affect the status epilepticus (SE) in rats. Furthermore, IL-1β, TLR4, HMGB1, P2X7 receptor, and EP2/PGE2 receptor antagonists have also been shown to modify SE in mice and rats (46). Lastly, a recent study in a mouse model of pentylenetetrazole-induced epileptic seizures also showed that administration of selenium nanoparticles had an anticonvulsant effect, which was mediated by decreased oxidative stress and neurotoxicity (47).

Due to the profound association between neuroinflammation and epileptogenesis, it is crucial to find diagnostic and monitoring biomarkers to provide biological insights on the role of inflammatory metabolism in neurological conditions and to develop novel diagnostic and therapeutic strategies to manage these disorders. In particular, cerebrospinal fluid (CSF) levels of neopterin, quinolinic acid, kynurenine, and tryptophan have been identified as potential biomarkers for neuroinflammation. Neopterin is a byproduct of the tetrahydrobiopterin *de novo* pathway synthesized by myeloid cells upon IFN-*γ* stimulation and it is found at increased levels in the CSF of individuals with neuroinflammatory disorders (48, 49). Furthermore, a preclinical study found increased levels of neopterin in the supernatant of primary rat astrocytes and mouse hippocampal slices following oxidative stress. These observations suggest that neopterin can also be produced by nerve cells under stress conditions (50). Another CSF biomarker of neuroinflammation is quinolinic acid (QA), a metabolite of the kynurenine pathway of tryptophan metabolism (49). QA has been implicated in the pathogenesis of neurological diseases in humans due to its potency as an excitotoxin. Elevated levels of QA can lead to oxidative stress, cytoskeletal disruption, behavioral alteration, and even cell death. At least some of the effects of QA can be attributed to its activation of the NMDA receptors (51). Kynurenine levels can also act as biomarkers for neuroinflammation and the activation of the kynurenine pathway of tryptophan metabolism can lead to immune suppression and neurotoxicity. Interestingly, it has been shown that modulation of this pathway can limit neurodegeneration in a murine multiple sclerosis model (52). Furthermore, the kynurenine/tryptophan ratio is used as a measurement for activity of indoleamine 2,3-dioxygenase, which is usually low under basal condition but can greatly increase during immune activation (49, 53). The downregulation of indoleamine 2,3-dioxygenase and the upregulation of hippocampal kynurenic acid lead to antiepileptic effects in a brain injury neonatal rat model of infantile spasms treated with antibiotics in combination with a ketogenic diet (54). Another recent study using a temporal lobe epilepsy rat model also showed reduced levels of tryptophan in the hippocampus and the anterior temporal lobe, which led to an enhanced frequency and amplitude of spontaneous excitatory postsynaptic currents in these brain regions (55). Because of their involvement in neuroinflammation, these metabolites can be used as reliable biomarkers in preclinical and clinical models. In particular, CSF neuropteran has an 82% sensitivity for defining neuro inflammation, followed by quinolinic acid with a 57% sensitivity, the kynurenine/tryptophan ratio with a 47% sensitivity, and finally kynurenine alone with 37% sensitivity in a pediatric cohort (49).

#### Clinical studies

In a study published by Hanin et al. involving 51 patients with new-onset refractory status epilepticus (NORSE), the levels of 12 cytokines/chemokines were measured in serum or cerebrospinal fluid (CSF). A comparison was made between patients with and without SE, as well as between the 51 patients with cryptogenic NORSE (cNORSE) and the 47 patients with known-etiology refractory status epilepticus (RSE).

Interestingly, The results showed a significant increase in the levels of IL-6, TNF-α, CXCL8/IL-8, CCL2, MIP-1α, and IL-12p70 pro-inflammatory cytokines/chemokines in patients with SE compared to those without SE, both in serum and CSF. Among patients with cNORSE, the serum levels of innate immunity pro-inflammatory cytokines/chemokines (CXCL8, CCL2, and MIP-1α) were significantly higher compared to patients with non-cryptogenic RSE (56).

In a separate study involving 85 children with idiopathic epilepsy, the concentrations of CSF neuron specific enolase (NSE), IL-1β, and EPO were measured. The epileptic groups showed a significant increase in the mean concentrations of CSF NSE, IL-1β, and EPO compared to the control groups (*P* < 0.01). Additionally, positive correlations were observed between the levels of IL-1β, NSE, and EPO (57).

Febrile infection-related epilepsy syndrome (FIRES) is a condition where individuals experience a NORSE following a febrile illness that occurred within two weeks to 24 h before the onset of refractory SE. In this case, febrile illness may or may not be accompanied by fever at SE onset. Seizures in FIRES are often resistant to treatment and can lead to long-term cognitive and neurological impairments. Understanding the underlying mechanisms and immune responses associated with FIRES is crucial for developing targeted therapeutic approaches. Kothur et al. have analyzed 32 cytokines and chemokines in CSF of pediatric patients with different epilepsy syndromes, including FIRES/FIRES-related disorders (FRD), febrile/afebrile status epilepticus (FSE and ASE), and chronic epilepsy with frequent daily seizures. Surprisingly, the elevation of such molecules was higher in FIRES, and in FSE, when compared to chronic epilepsy and controls without neurological or immunological disorders. Moreover, in FIRES Th1-associated cytokines and chemokines, as well as IL-6, CCL2, CCL19, and CXCL1, resulted elevated when compared to the levels observed in encephalitis, which involved a broader network of cytokines/chemokines. In FSE, CXCL9, CXCL10, CXCL11, and CCL19 were elevated compared to ASE, despite similar median seizure duration and timing of CSF testing in relation to seizures (58).

### Current treatments for status epilepticus and their effects on neuroinflammation

According to the American Epilepsy Society (AES) guidelines (59) published in 2016, the management of convulsive status epilepticus should start in the first 5 min with an early stabilization phase consisting in primary first aid for seizures based on the “ABC” approach, followed by administration of benzodiazepines (BDZs) (60–62).

About the role of BDZ in contrasting neuroinflammation, one of the first evidences in literature dates to 1996, when Park and colleagues demonstrated that BZDs could produce anti-inflammatory effects binding to microglial cells (63). According to this, Midazolam and Diazepam would be able to reduce the synthesis and release of proinflammatory and neurotoxic molecules generated by activated microglia (64) and to inhibit microglial activation and proliferation itself.

Moreover, diazepam seems to be able of inducing a state of cellular inactivation defining a reduced transcription factors activity and chemotactic aptitude, inhibition of Ca2C-mediated signaling and a diminished production of cytokines (65). If the seizures continue beyond 20 min, second-line therapy will be started.

Giving the lack of evidence about a better approach option, choices are often dictated by local availability, cost, and patient-specific factors. The options are IV phenytoin or Fosphenytoin, Valproic acid, or Levetiracetam (66). If none of the above suggested therapies are obtainable, IV phenobarbital could be considered (67).

Phenytoin can reduce the activation of m-TOR Pathway, decreasing the levels of proinflammatory cytokines such as IL-1β, IL-6 and TNF-α (68). VPA has antioxidant properties, inducing the suppression of lipid peroxidation and oxidative DNA damage, as well as anti-inflammatory effects, resulting in diminution of MPO permeation and microglial activation (69). It also promotes a decrease in brain inflammation and degeneration by regulation the NF-κB pathway (70) and discourages lipopolysaccharide-induced production of TNF-α and IL-6 (71, 72).

In the end, recent evidence suggests that Levetiracetam exerts neuroprotective effects via anti-inflammatory actions (73). It seems to be able to suppress the expression of proinflammatory molecules, such as TNF- α, IL-6 and IL-1β (74) and to reduce mononuclear phagocyte-mediated phagocytosis (75).

The third therapy stage should be contemplated when the seizure duration reaches 40 min and status epilepticus became refractory. Possibilities include repeating a second-line medication or resorting to an anesthetic drug (59).

Usually, the most used anesthetic agents include Midazolam, short-acting barbiturates (Pentobarbital/Thiopentone), and propofol. Currently, Midazolam is perhaps the most used due to faster onset of action and short duration of effect (76). Midazolam is recently reported to exert a neuroprotective effect by inhibiting inflammation. Through the regulation on the RhoA/ROCK2 pathway, it ameliorates the impairment of the blood–brain barrier against LPS (77). Then, based on recent findings, it is conceivable that midazolam might inhibit IL-1b-induced STAT3 phosphorylation and IL-6 release suppressing ROS production (78). However, further investigation will be required to clarify this concept.

According to some preclinical studies, use of anesthetics such as Propofol moderates the stimulation and minimizes the secretion of proinflammatory cytokines (79–81). Newly, Lu and colleagues validated that the favorable effects of Propofol are mediated by the JAK1/STAT3 way and that it could reveal anti-neuroinflammatory action by repression of proinflammatory mediators from microglial cells (82). However, its use in children should be limited because of the increased risk of Propofol infusion syndrome (PRIS), a life-threatening state characterized by rhabdomyolysis, arrhythmias, metabolic acidosis, myocardial and renal failure that can occur using doses greater than 65 mcg/kg/min for 48 or more hours (83).

As about one-third of the patients continue seizing despite these treatment lines, thus evolving to refractory SE, and half of these subsequently develop super-refractory SE, it seems important to consider further treatment alternatives (84).

Ketamine recently emerged as a promising treatment, due to advantageous hemodynamics and a singular mechanism of action than conventional anesthetics (85). Its great lipid solubility establishes a rapid CNS uptake and onset of action (86), moreover in late stages of SE, there is a decrease in the number of effective GABA-A receptors and up-regulated glutamate NMDA receptors that potentiate its action (87). It has also been shown that Ketamine may reduce neuroinflammation by diminishing the quantity of microglia and active macrophages in cerebral cortical tissues as well as TNF- α production (88, 89).

A variety of immunomodulatory treatments have been proposed over the past years. The most used include corticosteroids, IV immunoglobulin (IVIG) and plasmapheresis. Their application is sustained by modern findings on immunologic (antibodies against neural receptors such as voltage-gated potassium channels and NMDA receptors) and inflammatory (stimulation of inflammatory signaling pathways such as Interleukin-1 receptor/toll-like receptor pathway) actions that may provide to their basic pathophysiology (90–92).

The outcomes of these cures are inconstant, and researches are yet to have not confirmed a clear a certain efficacy response. They probably may be beneficial in recognized autoimmune epilepsies or entities with supposed immunological basis, such as febrile-infection related epilepsy syndrome (FIRES) (60), but further studies are needed necessaries.

Finally, therapeutic hypothermia could be pondered as an adjunctive therapy for RSE/SRSE (93). In fact, multiple case reports have shown its efficacy in resolution of RSE in children (94, 95) and this outcome seems to be due to its ability to diminish cerebral metabolic rate, cerebral edema, inflammation, oxidative stress, and glutaminergic inducement (96, 97).

### Innovative approaches to the management of neuroinflammation in patients with status epilepticus

Assuming its possible feasible role in the pathophysiology of epilepsy, targeting affecting the epileptogenic proconvulsant-convulsant effect of inflammatory cytokines appears as an innovative therapeutic option in drug resistant epilepsies and SEs. In this way we have the possibility of acting directly on the pathogenetic mechanisms rather than only on symptom control (98).

Many pharmacological studies involving IL-1β/IL-1R1, HMGB1/TLR4, COX-2/ prostaglandins or the complement system have revealed that these inflammatory pathways suggestively provide to the beginning and/or recurrence of SE and that their targeting may be potentially disease- modifying (99–103).

#### IL-1 beta blockade

The 2022 *International consensus recommendations for management of new onset refractory status epilepticustreatment* (104) supports the use of the human recombinant interleukin 1 (IL-1) receptor antagonist (Anakinra) in refractory SE.

Interleukin 1β (IL-1β) is a proinflammatory cytokine released by glial cells promoting neuroinflammation, enhancing neuronal excitability and contributing to refractories of seizures (105). Recent studies have shown that Anakinra could have a therapeutic role in controlling seizure recurrence and generalization in inflammatory refractory epilepsy (106–108), emerging as a suitable option for the treatment of SE of unknown cause in the early stages.

Moreover, the interleukin converting enzyme (ICE)/caspase-1, able to inhibit the conversion of pro-IL-1b to the pro-convulsant IL- 1b, has been recently contemplated as a possible target for the management of drug-resistant epilepsies (109). Pralnacasan (inhibitors of the IL-1b converting enzyme) and Belnacasan (elective inhibitor of caspases from the ICE/caspase-1 family) are currently undergoing phase III clinical trial, and the preliminary results are promising: In mice, intracerebroventricular administration of Pralnacasan and intraperitoneal administration of Belnacasan, appears to reduce 50% seizure duration (110, 111). Furthermore, a recent phase 2b double-blind randomized controlled trial involving the selective inhibitor of interleukin converting enzyme VX765, showed that in the 60 patients undergoing the therapy the percentage of responder-rate, of patients who were seizure-free for 2 weeks, and percentage of reduction in seizure rates ranged from 13% to 19% (112).

#### HMGB1-TLR4 axis

HMGB and TLR4 antagonists are also a potential novel anti-convulsive strategy (113, 114).

Resveratrol, for example, a type of natural phenol, has shown anti-inflammatory and neuroprotective properties (115). It has been recognized that it can suppress NFkB induced by TLRs 3 and 4 and the expression of INF-beta (116). Moreover, according to some research, this phenol reduces microglial activation and cyclooxygenases stimulation, often involved in epileptogenesis (117, 118). A study has also defined an antioxidant effect of Resveratrol against epileptogenic oxidative stress in the brain (119).

#### ATP-P2X7R signal

The ATP-gated purinergic P2X7 receptor (P2X7R) is an ion channel receptor situated on the superficial of microglia that can be activated by the ATP effluence following exposure to an exogenous stimulus, such as seizures (120–122). This stimulates the P2X7R-mediated NLRP3 inflammasome and the successive release of inflammatory molecules (123). According to recent data, demonstrating the defection in P2X7R expression after SE in both experimental animals and patients, the development of P2X7R antagonists could be useful in the treatment of refractory SE. Most reports (124–129) have suggested that Astaxanthin (AST), a molecule belonging to the carotenoid family, could be helpful in attenuating neuroinflammation in SE by inhibiting the ATP-P2X7R signal.

It has been demonstrated that it can reduce the extracellular ATP concentration, thereby constraining P2X7R activation and upregulation, causing the inhibition of the inflammatory signaling pathway. It can also considerably suppress the expression of inflammatory cytokine genes such as TNF-α, Cox-2, and IL-1β and it has strong antioxidant properties.

#### Interleukin-6 blockade

Increased serum and CFS levels of IL-6, an inflammatory cytokine having a pivotal role in enhancing and maintaining the inflammatory response and activating adaptive immunity, have been demonstrated in patients with refractory epilepsy (130, 131).

Supporting its involvement in these conditions, clinical and experimental data have reported some cases responding to treatment with Tocilizumab.

In 2018, Jun et al. (132) investigated the therapeutic potential Tocilizumab in 7 patients with new onset refractory status epilepticus (NORSE), reporting a resolution of SE after 1 or 2 doses of therapy in 6 patients with a median interval of 3 days from the initiation and no recurrence of SE during the observation period. Furthermore, in two children with Refractory Febrile Infection-Related Epilepsy Syndrome (FIRES), a decrease in seizures after Tocilizumab administration was documented with no side effects (133). The same favorable outcome was reported in a 6-year-old boy with Anakinra-Refractory FIRES (134).

#### Ketogenic diet

Ketogenic diet (KD) represents a promising approach: it is a therapeutic dietary characterized by low-calorie, low-carbohydrate, high-fat, and standard protein intake which collectively sustain a state of ketosis, closely resembling the metabolic state induced by fasting.

Although literature data about its anti-inflammatory properties are poor, recent studies (135) have shown that it may influence neurotransmitter levels related to seizure onset. Specifically, the KD appears to enhance the activity of the inhibitory neurotransmitter gamma-aminobutyric acid (GABA). This effect is achieved through different pathways, including the activation of glutamic acid decarboxylase and the inhibition of transaminase activity. Furthermore, the KD could elevate the epileptic threshold by increasing ATP-sensitive potassium channels (136, 137). Beyond these seizure-related benefits, the KD may also exert a neuroprotective action. This includes the upregulation of calbindin, the inhibition of apoptotic factors like caspase 3, and an increase in the concentration of kynurenic acid. Additionally, the diet reduces the presence of oxygen free radicals (ROS) through the elevation of polyunsaturated fatty acids and neuronal uncoupled proteins (138–140).

Moreover, several studies have consistently observed that the ketogenic diet (KD) influences the diversity of the microbiome, leading to changes in the production of gut metabolites. In the context of epilepsy, it is noteworthy that children with this condition exhibit alterations in their gut microbiota, potentially contributing to the development or severity of seizures. Some research has postulated that changes in the expression of short-chain fatty acids (SCFA), which are gut metabolites capable of crossing the blood-brain barrier, may provide valuable insights into the modulatory effects of the KD on certain diseases. However, the precise mechanisms through which SCFA may influence disease expression remain to be fully elucidated (138, 141).

#### Cannabidiol

The management of epilepsy with cannabidiol (CBD), a cannabis derivative, has engendered impressive enthusiasm in recent years.

Numerous experimental reports indicate that CBD can diminish seizure occurrence and length with standard and unconventional antiepileptic properties, along with a neuroprotective and anti-inflammatory function (142). Several preclinical studies suggested that CBD revealed strong inhibitory effects of neurotoxic molecules and inflammatory cytokines, emphasizing its therapeutic potential for the treatment of refractory SE (143). Although CBD's effects on neuroinflammation appear to be still poorly understood, its molecular mechanism of action seems to be related to the downregulation of NADPH oxidase-mediated ROS, TLR4-NF*κ*B and IFN-*β*-JAK-STAT pathways (144, 145). Gofshteyn et al. (142) reported the potential therapeutic effect of cannabidiol for FIRES in a series of 7 pediatric patients. Moreover, Rajsekar R. Rajaraman et al. (146) described the situation of a child with long-standing super-refractory status epilepticus (SRSE) who exhibited quick and complete resolution of SRSE upon exposure to pure cannabidiol. These evidences suggest that CBD can be considered as a potential treatment in SRSE.

### Targeting neuroinflammation in patients with status epilepticus

The involvement of the inflammatory response in SE led to the search for therapeutic strategies targeting inflammatory mediators in this condition (2, 147). Currently, clinical experience is limited to the use in isolated case reports or series of the anti-cytokine agents Anakinra and Tocilizumab (148). However, this field is in continuous expansion, since studies on animal models of epilepsy and SE have led to the identification of potential new therapeutic targets.

Both Anakinra and Tocilizumab are currently used in the treatment of several autoinflammatory disorders (such as familial Mediterranean fever), other rheumatic diseases (arthritis, vasculitis), and cytokine-release syndromes (CRS) (3, 149, 150).

In SE, the most well-recognized application of Anakinra is the administration in patients with the febrile infection-related epilepsy syndrome (FIRES), and other reports describe its use in new-onset refractory status epilepticus (NORSE) or refractory/super refractory status epilepticus (SRSE) (148) To date, in the published cases Anakinra has been administered with different regimens (timing for drug initiation, posology, treatment duration) and resulted in a clinically relevant reduction of seizures in more than half of the reported patients (148, 151–154), while data on the long-term neuropsychological outcome have to be better defined7,10. Concerning the safety profile of Anakinra in epileptic individuals, the occurrence of infections has been reported in about 30% of the patients, but drug withdrawal due to severe adverse events is only rarely reported (148, 151–154).

The experience with the use of Tocilizumab in SE is limited to less than 50 patients, mostly suffering from NORSE or SRSE. Similarly to Anakinra, Tocilizumab has shown a clinical effect of reduction/arrest of seizures in most of the patients, while the safety profile showed the development of clinically relevant infections in about 20% of the described patients (132, 133, 148, 153, 155).

Interestingly, Tocilizumab has been effectively used to treat a patient with COVID-19-associated SE, as well as patients with CRS following chimeric-antigen T cell receptor (CAR-T) cells administration (156, 157).

Although the role of Anakinra and Tocilizumab in SE is promising, the interpretation of their efficacy is complicated by the heterogeneity of the available studies, together with the frequent concomitant administration of other treatments (immunosuppression, ASMs). Also, the use of biomarkers, such as the analysis of serum and CSF cytokines, has been performed only in a reduced percentage of patients, and the correlation between their trend and the clinical outcome is not defined (148).

The role of other biologic agents, including the anti- TNF- α Etanercept and the anti-CD20 antibody Rituximab, is limited to patients with specific etiologies of epilepsy and SE. Targeting TNF- α represents a promising strategy for patients suffering from Rasmussen encephalitis, in which the administration of Etanercept has shown a decrease in seizure frequency, although there are no specific data regarding its use in SE (158, 159). On the other hand, the application of Rituximab is limited to patients with drug-resistant epilepsy in the context of autoimmune encephalitis (160), while the use in cryptogenic RSE did not provide a clinically relevant effect in a recent small case series published by Jun et al. (132). Regarding therapies targeting integrins, a recent trial on the administration of the anti-α4-integrin antibody Natalizumab evidenced a reduction in the seizure frequency in patients with drug-resistant epilepsy (161), although the drug has not yet been investigated in SE.

## Conclusions

**Figure 1:**
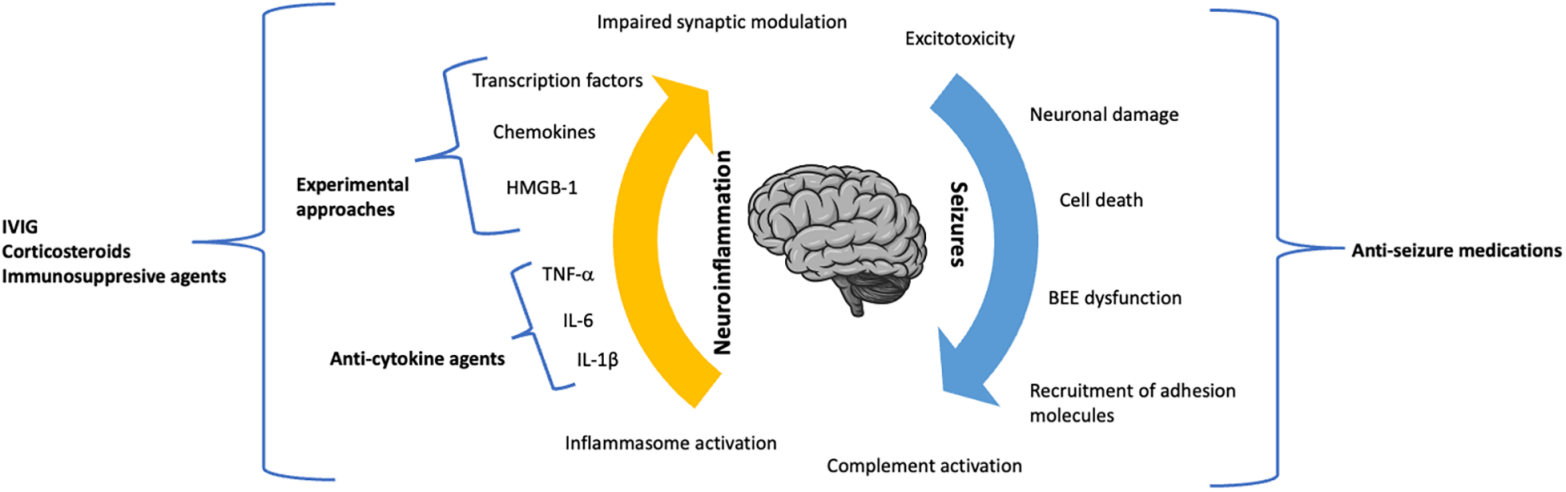
The dynamic role of neuroinflammation and seizure activity in the pathogenic process of status epilepticus.

SE represents a hard therapeutic challenge, and there is currently no standard approach to refractory or super-refractory cases. The use of animal models of SE, as well as the *in vivo* determination of serum and inflammatory biomarkers and the analysis of bioptic/surgical specimens have pointed out the central role of neuroinflammation in initiating and perpetrating the pathogenic process leading to SE. Therefore, there is an urgent need to develop therapeutic strategies targeting neuroinflammation in this category of patients. Although the clinical experience with drugs targeting neuroinflammation in SE is currently limited to the use of anti-IL-1 and anti-IL-6 agents, preclinical research is rapidly progressing and will hopefully lead to the identification of new targeted therapies (Figure 1), including chemokines and their receptors, and intracellular signaling molecules.
